# Pre-Vaccination COVID-19 Vaccine Literacy in a Croatian Adult Population: A Cross-Sectional Study

**DOI:** 10.3390/ijerph18137073

**Published:** 2021-07-02

**Authors:** Ivana Gusar, Suzana Konjevoda, Grozdana Babić, Dijana Hnatešen, Maja Čebohin, Rahela Orlandini, Boris Dželalija

**Affiliations:** 1 Department of Health Studies, University of Zadar, 23000 Zadar, Croatia; igusar@unizd.hr (I.G.); grozdana.babic@gmail.com (G.B.); bdzelalija@unizd.hr (B.D.); 2 Faculty of Medicine, Josip Juraj Strossmayer University of Osijek, 31000 Osijek, Croatia; hnatesen.dijana@kbco.hr (D.H.); maja.cebohin@skole.hr (M.Č.); 3 General Hospital Zadar, 23000 Zadar, Croatia; 4 Clinical Department of Pain Management, University Hospital Osijek, 31000 Osijek, Croatia; 5 Nursing Institute “Professor Radivoje Radić”, Faculty of Dental Medicine and Health Osijek, Josip Juraj Strossmayer University of Osijek, 31000 Osijek, Croatia; 6 Medical School Osijek, 31000 Osijek, Croatia; 7 Department of Health Studies, University of Split, 21000 Split, Croatia; rahela.orlandini@ozs.unist.hr; 8 School of Medicine, University of Split, 21000 Split, Croatia

**Keywords:** COVID-19, vaccine literacy, vaccination, pandemic, general population

## Abstract

Despite world-level efforts and the endeavors of scientists and medical professionals in suppressing the COVID-19 pandemic, inadequate levels of vaccine literacy of the general population can represent a grave obstacle. The aim of this study was to evaluate COVID-19 vaccine literacy in the Croatian adult general population before vaccination began. The specific objectives were to test differences regarding socio-demographic characteristics and to examine perceptions and attitudes about vaccination against COVID-19 considering the level of VL against COVID-19. A cross-sectional study with a translated and psychometrically tested questionnaire was conducted in 1227 participants before the start of vaccination, from 15 to 31 January 2021. The results show a medium level of vaccine literacy (M = 2.37, SD = 0.54) and a significant difference between functional and interactive-critical vaccine literacy (*p* < 0.001). The level of vaccine literacy grew with the level of education (*p* = 0.031) and reduced with age (*p* < 0.001). Participants who were employed, had chronic diseases, took medicine, or consumed alcohol daily had a lower level of vaccine literacy. There is room for progress in the COVID-19 VL level for the adult population in Croatia, especially at the interactive-critical VL, which could have an important role in people accepting the vaccine against the COVID-19 disease. A satisfactory level of vaccine literacy in the population is necessary because it can contribute to the fight against the pandemic.

## 1. Introduction

The pandemic caused by the coronavirus is a major public health challenge that the world has recently confronted [1,2,3]. Due to its fast spread, not long after the infection had appeared in China at the end of 2019, Croatia was also added to the list of countries affected by the pandemic in February of 2020 [4]. As the number of infected grew, discussion regarding the necessity of stronger prevention arose, which was not only about introducing additional restrictions in behavior and habits of residents but also about commencing immunization. As in other countries around the world [5,6], the need for immunization in Croatia is also highly covered by the media. In addition to the adjustment and sensitization of the general population, the media could also cause negative effects [7], since not only is true information provided, but false information is provided as well, which is a great threat to public health [8,9]. In this extraordinary period, besides health and economic systems, the pandemic also reflects on the social system of community. Therefore, health literacy of the general population is exceptionally important [10].

Health literacy is defined as a personal, cognitive, and social skill that determines the capability of an individual to access, understand, and use information to improve and maintain personal health [11]. The concept and definition of health literacy are directly connected to health education, whose focus is the ability of an individual to understand and, in the case of having to make a decision, to use health information efficiently [7]. People who have a satisfactory level of health literacy manage their health more efficiently [2]. When it comes to types of health literacy, there are functional, interactive, and critical health literacy [12]. Functional health literacy refers to basic reading and writing skills, i.e., language/linguistic skills, including the comprehension of read content [13,14]. Interactive health literacy, also called communicative literacy, implies advanced skills that include cognitive efforts, such as solving problems and making decisions [13,14]. Furthermore, critical health literacy implies the highest level of cognitive and social skills, a critical analysis of health information, an improvement in individual and social capacities, and an understanding of the political and economic dimensions of health [12,13]. So far, research accentuates how individual and social factors affect the level of health literacy of an individual. Moreover, the majority of conceptual models precisely identified personal factors and characteristics of the public health system as the factors that influence the level of health literacy [14]. Nonetheless, during the 1990s, studies showed an interconnection between health literacy and the health of an individual, pointing out a correlation between a lower literacy rate and a poorer/weaker health status [15]. Moreover, the elderly and people with lower education levels are recognized as risk groups, while gender has been shown in previous research to have differing levels of risk [13].

The concept of vaccine literacy (VL) is founded on the concept of health literacy [7] and is defined not only as a level of knowledge about vaccination but also as the development/construction of a system that would facilitate the communication or spread of messages about vaccines as being necessary, without which the functioning of the health system would be impossible [7]. Bauer and others (2017) conducted research about vaccine rejection in Austria, in which they explained how generally a very low level of knowledge is present about vaccination, along with an accentuated fear of the consequences of vaccination [16]. The authors see a potential solution in the implementation of specific education in the early education of children and in raising the awareness of doctors as the most reliable source of health information [16]. The majority of previous research was done in America, and those authors did not establish a clear connection between health literacy level and the decision about vaccination of an individual [17]. American scientists recognize the current global pandemic as a convenient way of promoting vaccination and building resistance to misinformation about COVID-19 disease and vaccination [6]. Given the prevalence of the COVID-19 disease, circumstances caused by the pandemic, and the importance of acceptance of vaccination that will protect the population or at least prevent severe disease caused by this virus, it is important to research the level of VL in the population and examine the potential contribution of VL when accepting the vaccination. Therefore, the aim of this study was to evaluate COVID-19 VL in the Croatian adult general population before vaccination began. The specific objectives were to test differences regarding socio-demographic characteristics and to examine perceptions and attitudes about vaccination against COVID-19, considering the level of VL against COVID-19.

## 2. Materials and Methods

A cross-sectional study was conducted before vaccination against COVID-19 disease, starting in the period from 15 to 31 January 2021. Due to the epidemiological limitations, the authors of this paper sent to participants Email, Facebook, and Instagram contacts a Google forms survey link with a detailed explanation of the study and a request that they participate, as well as a request that they further disseminate the questionnaire to potential participants older than 18 years. This combination of different social networks was used to ensure greater circulation of the questionnaire and the representation of different groups of participants [18]. Additionally, the first questionnaires sent were balanced according to different geographical regions in Croatia. After reading the instructions, the participants (>18 years of age) could choose to complete the questionnaire or not.

In the research, 1227 participants were surveyed, all older than 18 years. There were 935 women (76.2%) and 292 (23.8%) men from all 21 counties of Croatia. More than half of them, 780 (63.6%), belonged to the group aged from 18 to 34 years. More than half of the participants were employed (767; 62.5%), while few of them were retired (74; 6.0%). Amongst them, there were 219 people (17.8%) who had had COVID-19, and there were 433 people (35.3%) that had been in isolation or self-isolation. Out of those 433, 69 (5.6%) of them had been isolated or self-isolated more than once. All data are shown in Table 1.

In this research, a translated questionnaire regarding COVID-19 VL, to which the author has previously consented, was used [13]. The instrument was translated to Croatian by two independent experts. The experts have fulfilled the terms of competences for the translation [19]. The final Croatian translation resulted in the agreement of both experts, after which the instruments were reviewed by a professor of Croatian linguistics. Back-translation of the instruments to English was done by the professor, who did not participate in previous translations. The authors verified the synonymy of the back-translation version and the original instruments. According to the age and level of education, the participants were divided into six groups and according to the employment status into employed, unemployed and retired. Before applying the instruments, four people of all age groups (of ages 19, 25, 40, and 68) reviewed and positively evaluated the comprehensibility of the final versions of the instruments. Principal component analysis (PCA) was conducted to test the factor structure, which is presented in Table 2 and Supplementary Materials Table S1. The internal consistency of the scale was assessed trough Cronbach’s alpha coefficient, and it resulted in a value of 0.81 for the whole questionnaire. The COVID-19 VL questionnaire contains 12 items in total with two factors. One factor contains four items and refers to assessing functional VL. The second has eight items to measure interactive-critical VL. Each response has a 4-point Likert scale (from 1 never to 4 often, and in reversed items from 1 often to 4 never), and a higher value corresponds to a higher VL level (13). Moreover, data about health conditions and health habits were collected, along with the socio-demographic characteristics of participants, as shown in Table 1.

Statistical analysis of the acquired results was performed using the Statistica 13 computer application (TIBCO Software Inc., Palo Alto, USA 2018). Statistical analysis was performed in two steps. In the first step of processing of the results, the latent structure of the applied questionnaire was checked using PCA. The reliability of the questionnaire was verified by Cronbach’s alpha coefficient. In the further analysis, the descriptive data (arithmetic mean, standard deviation, and percentages) were calculated. Due to the normal distribution of the results tested by the Kolmogorov–Smirnov test, during the testing of the differences among the participants, the *t*-test was used.

The ethical committee of the University of Zadar approved the research (protocol code: 114-06/21-01/03; number: 2198-1-79-37/21-02; 13 January 2021). All participants were informed of the aim of the research and voluntarily agreed to participate in the research. The anonymity of participants during and after the research was guaranteed.

## 3. Results

### 3.1. COVID-19 VL

The participants had a mean level of COVID-19 VL of 2.37 (SD = 0.54). That is, the level regarding the functional mean score was M = 2.86 (SD = 0.71), while the level of the interactive-critical mean was M = 2.12 (SD = 0.75). The results indicate significant differences between functional and interactive-critical literacy (*t* = 25.082; *p* < 0.001).

### 3.2. COVID-19 VL Considering Socio-Demographic Characteristics

**Figure 1:**
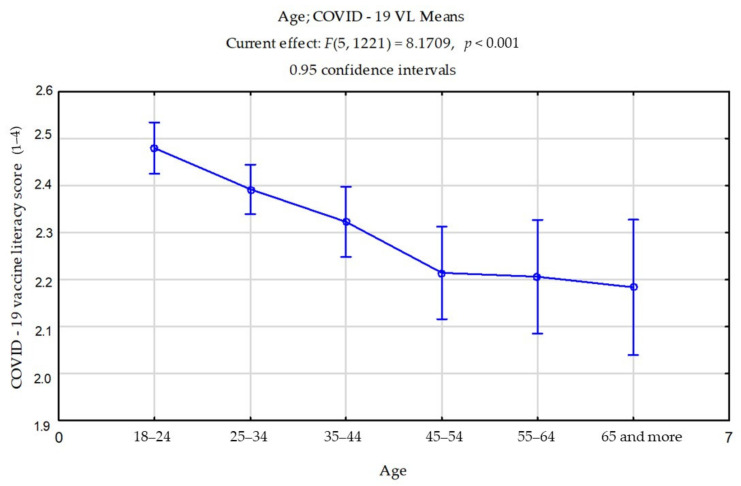
COVID-19 VL level considering age (*n* = 1227).

When it comes to socio-demographic characteristics of participants, there was no statistically relevant difference in COVID-19 VL considering gender (*t* = 0.010; *p* = 0.991). However, there was a statistically relevant difference considering age (Figure 1; Table 3).

The level of COVID-19 VL significantly increased with the level of education (*t* = 2.453, *p* = 0.032). Employed participants had a M = 2.34 (SD = 0.53) COVID-19 VL level, which was significantly less (*t* = −3.698; *p* < 0.001) compared with the unemployed M = 2.47 (SD = 0.55). However, it was significantly higher (*t* = 2.580; *p* = 0.010) among retired participants M = 2.17 (SD = 0.52). Referring to other characteristics of participants, no significant difference was determined in general COVID-19 VL, but there was a significant difference in the functional VL for participants who were not chronically ill and those who did not consume alcohol. Participants that consume/take medicine on a daily basis had a significantly lower level of COVID-19 VL, i.e., functional VL (*t* = 2.644; *p* = 0.008) and interactive-critical VL (*t* = −2.529; *p* = 0.011), in comparison with other participants. Participants who had not received all mandatory vaccines had a significantly higher level (*t* = −3.028; *p* = 0.002) of interactive-critical skills M = 2.34 (SD = 0.72) in comparison with other participants who received all mandatory vaccines. There was no significant difference in the level of COVID-19 VL between participants who had had COVID−19 and those who had not (*t* = 0.776; *p* = 0.437). However, a significantly higher level of COVID−19 VL was determined by comparing participants who had not had COVID-19 with participants who did not know if they had ever had COVID-19 (*t* = −2.062; *p* = 0.039), as well as in comparison with participants who had had COVID-19 (*t* = −3.482; *p* = 0.000). There was no significant difference (*t* = −0.857; *p* = 0.391) in the level of COVID-19 VL among participants in self-isolation.

### 3.3. Attitudes and Perceptions about Future COVID-19 Vaccines

Further data processing referred to testing differences in perception and opinions of participants about vaccination against the COVID-19 virus considering the level of their functional and interactive-critical VL. Results presented in Table 4 show a statistically significant difference in the perception and opinions of participants considering the level of functional and interactive-critical COVID-19 VL.

Participants who answered the questions affirmatively had a higher level of functional and interactive-critical COVID-19 VL.

## 4. Discussion

We live in a time that will enter history as an unforgettable health crisis of world proportion, marked not only by competent information but also by an explosion of often inaccurate information via the internet and social media [2,8]. Therefore, the goal of this research was to evaluate COVID-19 VL in the Croatian adult general population before vaccination began and to examine the perceptions and attitudes about vaccination against COVID-19 considering the level of VL about COVID-19.

Although some previous research examined only functional VL [17], our research was conducted using a questionnaire that examined functional and critical-interactive VL. Using both subscales provides not just an insight into the knowledge of the participants regarding their understanding of the information but also an insight into the attitudes of the participants about the information received. Although our research sample did not fully correspond to the characteristics of the general population in Croatia, our results point out an average level of COVID-19 VL. However, participants have a significantly higher level of functional VL, which represents basic language skills and reading comprehension, in comparison with interactive-critical VL, which includes critical analysis and effective use of health information. In other words, we can conclude that participants can better understand content connected to the COVID-19 disease than they can use the received information critically. The desired level of VL implies cognitive abilities, such as comprehension, problem solving, and decision making [13]. Comparing this research to the research done in Italy in June 2020, our participants had lower levels of both functional and interactive-critical VL, which can be explained by the fact that Croatia was affected by the pandemic sometime after Italy was [20], so the Croatian population had less information about, and less interest in, the COVID-19 virus.

Furthermore, the results of our research did not show a significant difference in the level of functional and interactive-critical COVID-19 VL regarding gender. In their review article, Sorensen et al. [14] highlighted gender as a personal predicative determinant for health literacy, as well as the fact that results of previous research are inconsistent. Results of this research show significant changes in the level of VL considering the age of participants. It is noticeable that the level of COVID-19 VL decreases as age increases. However, data point to a difference in the range of levels of COVID-19 VL amongst elderly participants, while the participants of younger age groups were at similar levels of knowledge. These results could be a consequence of differences in the education level among older participants. Previous research also recognized age as an important factor in the level of health literacy [13,14,21]. Furthermore, previously mentioned research done in Italy showed similar levels of VL depending on the participant’s age [13]. As established in previous relevant research, the higher the level of education was, the higher the level of COVID-19 VL was [2,13,15,16]. Employed participants had a significantly lower level of COVID-19 VL in comparison with unemployed participants, which is in opposition to previous research [22]. However, Khoshravesh et al. [23] also reported about the borderline level of health literacy of employed people in Iran. Other authors also point out that employment is a significant factor in the level of health literacy [14]. The results of our research may be a direct consequence of the amount of time that unemployed people spend exposed to information from mass media. We assume that unemployed people at the time of restrictive measures spent most of their time at home, following the news and consuming large amounts of information via media [6]. However, COVID-19 VL levels of employed people were significantly higher in comparison with retired participants, which is, we assume, a consequence of the previously mentioned changes in the levels of COVID-19 VL with the increase in the age of the participants and the possible differences in education level. We assume that older participants differ from each other with regard to education level, which suggests there will be a difference between retired people who are older and have a higher level of education than other old people. Participants who were not chronically ill and did not consume alcohol had a significantly higher level of functional COVID-19 VL in comparison with participants who were chronically ill and consumed alcohol daily. This result could also be a consequence of the older age of the participants and the fact that chronic illness is more frequent as age increases [24], along with a more frequent consumption of alcohol [25]. Since the differences in level of VL depend on age, it is possible to draw connections regarding the lower results of participants who consumed medicine daily as well, since those participants were elderly [26]. Our results point to significant differences between participants who had not received all mandatory vaccinations and those who had, where those who had not received them had higher levels of interactive-critical COVID-19 VL. Biasio showed the same data and explained how we risk efficient evaluations of received information and make the wrong decisions when too much information is received [7]. Sometimes people with a satisfactory level of health literacy can make incorrect estimations because they are exposed to too much information [7]. Furthermore, Aharon and others published research done on 731 parents of children aged 3–4, concluding as well that higher levels of health literacy result in higher instances of not receiving the recommended vaccinations, and higher levels of interactive-critical literacy were correlated with lower vaccination rates [27]. However, Spring accentuates that health literacy empowers people and communities to participate in personal health protection, improves health and benefits, and solves health inequalities by building the resistance of an individual and community [2]. Participants who were uncertain whether or not they had had COVID-19 were significantly different from participants who were certain about it. This result was, we assume, a consequence of the high personal interest of participants in information that was widely available since the pandemic began [2,7].

Our results that present perceptions and attitudes about vaccination against COVID-19 considering the levels of literacy about COVID-19 showed that participants with significantly higher levels of VL more often provided affirmative answers to questions about the production of safe and efficient vaccines, about their personal responses to vaccination, about the possibility of vaccinating the entire population, and the need to vaccinate children. These results were in accordance with the results of studies (done) in Italy. Participants with higher levels of functional and interactive-clinical COVID-19 VL responded affirmatively in higher percentages. It is assumed that these data are evidence of a reliance among the general population in Croatia on health and scientific professions, which has been previously confirmed by other research [8,28].

Our sample was exclusively internet based and as we previously indicated do not fully correspond to the characteristics of general adult population in Croatia. We find that the uneven distribution of participants within different age groups is deficient, which certainly affected the distribution of participants in educational levels, as well as other categories. This distribution was probably conditioned by the method of data collection and the fact that elderly people use IT equipment less often, therefore participating less frequently in online research. The above suggests the need for future implementation of combined research that would be conducted online but also by paper-and-pencil method, as recommended by Regmi et al. [18]. Moreover, these data were collected at a time when many other studies on COVID-19 illness were performed, which could cause population saturation and a lower response among potential participants. Finally, this was a cross-sectional study whose results cannot yield causal relations, which also represents a certain restriction in the interpretation of collected data.

To the best of our knowledge and review of the available literature, this is the first study in our region that can improve current knowledge of the general population’s attitudes toward vaccination against COVID-19. Although these data were collected prior to vaccination, they may indicate the direction and necessary activities that will ensure an increase in general public awareness of the importance of accepting the COVID-19 vaccine.

## 5. Conclusions

There is room for progress in the level of COVID-19 VL among the adult population in Croatia, especially in interactive-critical VL, which could play an important role in accepting the vaccine against the COVID-19 disease. The results suggest the possibility that different forms of employment and education level in all age groups have effect on accepting the COVID-19 vaccine. Considering this, public health institutions should find an effective means of education and especially focus on the education of elderly people with low education level and people with additional comorbidities, both of whom are the most endangered. Satisfactory levels of COVID-19 VL among the population, which are now a high priority in public health activities, are necessary and extremely important because high levels of VL and vaccination can make a significant contribution in fighting against the ongoing pandemic.

## Figures and Tables

**Table 1:** Demographic characteristics of the participants (*n* = 1227).

Variable		Number (*n*)	Percentage (%)
Gender	Males	292	23.8
	Females	935	76.2
Age	18–24	374	30.5
	25–34	406	33.1
	35–44	202	16.5
	45–54	115	9.4
	55–64	76	6.2
	≥65	54	4.4
Education	Elementary school	30	2.4
	High school	517	42.1
	Undergraduate	277	22.6
	Graduate	331	27
	Master	62	5.1
	Doctorate	10	0.8
Occupational status	Employed	767	62.5
	Non-employed	386	31.5
	Retired	74	6
I suffer from a chronic illness.	Yes	227	18.5
	No	1000	81.5
I use medication daily as recommended by my doctor.	Yes	551	44.9
	No	676	55.1
I consume tobacco products daily.	Yes	387	31.5
	No	840	68.5
I consume alcohol products daily.	Yes	49	4
	No	1178	96
I have been properly vaccinated so far.	Yes	1128	91.9
	No	99	8.1
I have had COVID-19.	Yes	219	17.8
	No	702	57.2
	I do not know	306	25
I have been in self-isolation.	Yes	364	29.7
	No	794	64.7
	Yes, multiple times	69	5.6

**Table 2:** Factor Loading—PCA (*n* = 1227).

Items	Factor 1	Factor 2
1	0.142463	**−0.829147**
2	0.118460	**−0.879581**
3	0.168398	**−0.853559**
4	0.134923	**−0.813179**
5	**−0.650464**	−0.224153
6	**−0.686086**	0.031209
7	**−0.689637**	−0.028863
8	**−0.667444**	−0.145471
9	**−0.777003**	−0.142782
10	**−0.840424**	−0.063800
11	**−0.830299**	−0.065123
12	**−0.765684**	−0.020569

bold—the greatest correlation.

**Table 3:** COVID-19 functional and interactive-critical VL level considering age (*n* = 1227).

Age	*n* (%)	Functional Mean Score * (SD)	*p* **	Interactive-Critical Mean Score * (SD)	*p* **	Vaccine Literacy Total Score *	*p* **
18–24	374 (30.5)	2.95 (0.69)		2.24 (0.78)		2.47 (0.57)	
25–34	406 (33.1)	2.98 (0.68)		2.09 (0.78)		2.39 (0.54)	
35–44	202 (16.5)	2.77 (0.70)	<0.001	2.09 (0.69)	<0.001	2.32 (0.51)	<0.001
45–54	115 (9.4)	2.74 (0.51)		1.95 (0.61)		2.21 (0.46)	
55–64	76 (6.2)	2.69 (0.69)		1.96 (0.68)		2.20 (0.49)	
≥65	54 (4.4)	2.21 (0.77)		2.16 (0.78)		2.18 (0.54)	

* possible range from 1 to 4; ** *t*-test.

**Table 4:** Attitudes and perceptions about future COVID-19 vaccines and VL.

Variable	VL Functional Score					VL Interactive-Critical Score				
	A *	Mean (SD)	*t*	df	*p* *	A*	Mean (SD)	*t*	df	*p* **
Will it be possible to produce safe and efficacious vaccines?	Yes	2.86 (0.71)	63.25	2452	<0.01	Yes	2.12 (0.75)	31.40	2452	<0.01
	No	1.32 (0.46)				No	1.32 (0.46)			
Will you get vaccinated, if possible?	Yes	2.86 (0.71)	53.67	2452	<0.01	Yes	2.12 (0.75)	22.82	2452	<0.01
	No	1.53 (0.49)				No	1.53 (0.49)			
Will authorities succeed in vaccinating the entire population?	Yes	2.86 (0.71)	44.83	2452	<0.01	Yes	2.12 (0.75)	11.82	2452	<0.01
	No	1.83 (0.36)				No	1.83 (0.36)			
Would you pay a fee to be vaccinated?	Yes	2.86 (0.71)	47.01	2452	<0.01	Yes	2.12 (0.75)	15.21	2452	<0.01
	No	1.74 (0.43)				No	1.74 (0.43)			
Should children be vaccinated too?	Yes	2.86 (0.71)	46.74	2452	<0.01	Yes	2.12 (0.75)	14.83	2452	<0.01
	No	1.75 (0.43)				No	1.75 (0.43)			

* Answers; ** *t*-test.

## Data Availability

The data presented in this study are available upon request from the corresponding author.

## Supplementary Materials

The following are available online at https://www.mdpi.com/article/10.3390/ijerph18137073/s1, Table S1. Factor Loading—PCA.
