# Supplementary material for: Pre-Vaccination COVID-19 Vaccine Literacy in a Croatian Adult Population: A Cross-Sectional Study
Source: Int J Environ Res Public Health. 2021 Jul 2;18(13):7073. doi: 10.3390/ijerph18137073 (PMC8297136; doi:10.3390/ijerph18137073)
Supplement: Supplementary file 1 [file ijerph-18-07073-s001.zip › ijerph-1243162-supplementary.pdf]

Table S1. Factor Loading—PCA

Variable (English/Croatian)	Items (English/Croatian)	Factor 1	Factor 2
When reading or listening to information about future COVID-19 vaccines or current vaccines: / Kada čitate ili slušate informacije o trenutnim ili budućim cjepivima protiv COVID-19:	1. Did you find words you did not know? / Jeste li naišli na riječi koje niste znali?	0.142463	**−0.829147**
	2. Did you find that the texts were difficult to understand? / Jeste li naišli na tekst koji ste teško razumjeli?	0.118460	**−0.879581**
	3. Did you need time to understand them? / je li Vam trebalo puno vremena za razumijevanje teksta?	0.168398	**−0.853559**
	4. Did you or would you need someone to help you understand them? / Bi li Vam trebao netko tko će Vam pomoći u razumijevanju?	0.134923	**−0.813179**
When looking for information about future COVID-19 vaccines or current vaccines: / Kada tražite informacije o trenutnim ili budućim cjepivima protiv COVID-19:	5. Have you consulted more than one source of information? / Razmatrate li u više od jednog izvora informacija?	**−0.650464**	−0.224153
	6. Did you find the information you were looking for? / Jeste li pronašli informacije koje ste tražili?	**−0.686086**	0.031209
	7. Have you had the opportunity to use the information? / Jeste li imali priliku koristiti informacije?	**−0.689637**	−0.028863
	8. Did you discuss what you understand about vaccinations with your doctor or other people? / Jeste li sa svojim liječnikom ili nekim drugim ljudima (laicima) razgovarali o onome što ste razumjeli o cijepljenju?	**−0.667444**	−0.145471
	9. Did you consider whether the information collected was about your condition? / Jeste li razmotrili tiču li se prikupljene informacije i Vašega stanja?	**−0.777003**	−0.142782
	10. Have you considered the credibility of the sources? / Jeste li razmotrili vjerodostojnost izvora?	**−0.840424**	−0.063800
	11. Did you check whether the information was correct? / Jeste li provjerili točnost podataka?	**−0.830299**	−0.065123
	12. Did you find any useful information with which to make a decision on whether or not to get vaccinated? / Jeste li pronašli korisne informacije za donošenje odluke hoćete li se cijepiti ili ne?	**−0.765684**	−0.020569

bold - the greatest correlation.
